# Multicellular 3D Models to Study Tumour-Stroma Interactions

**DOI:** 10.3390/ijms22041633

**Published:** 2021-02-05

**Authors:** Elisabetta Colombo, Maria Grazia Cattaneo

**Affiliations:** Depterment of Medical Biotechnology and Translational Medicine, Università degli Studi di Milano, 20129 Milano, Italy; elisabetta.colombo10@studenti.unimi.it

**Keywords:** 2D cell culture, 3D cell culture, multicellular spheroids, tumor microenvironment, TME, extracellular matrix, ECM, cell–cell communication, CCC

## Abstract

Two-dimensional (2D) cell cultures have been the standard for many different applications, ranging from basic research to stem cell and cancer research to regenerative medicine, for most of the past century. Hence, almost all of our knowledge about fundamental biological processes has been provided by primary and established cell lines cultured in 2D monolayer. However, cells in tissues and organs do not exist as single entities, and life in multicellular organisms relies on the coordination of several cellular activities, which depend on cell–cell communication across different cell types and tissues. In addition, cells are embedded within a complex non-cellular structure known as the extracellular matrix (ECM), which anchors them in a three-dimensional (3D) formation. Likewise, tumour cells interact with their surrounding matrix and tissue, and the physical and biochemical properties of this microenvironment regulate cancer differentiation, proliferation, invasion, and metastasis. 2D models are unable to mimic the complex and dynamic interactions of the tumour microenvironment (TME) and ignore spatial cell–ECM and cell–cell interactions. Thus, multicellular 3D models are excellent tools to recapitulate in vitro the spatial dimension, cellular heterogeneity, and molecular networks of the TME. This review summarizes the biological significance of the cell–ECM and cell–cell interactions in the onset and progression of tumours and focuses on the requirement for these interactions to build up representative in vitro models for the study of the pathophysiology of cancer and for the design of more clinically relevant treatments.

## 1. Rise and Fall of 2D Cell Cultures

Knowledge in cellular biology has constantly been improved as a result of the concurrent development and accessibility of innovative reagents and techniques. It was the setup of first rudimentary microscopes that allowed Robert Hooke (1635–1703) to originally observe the small compartments of a slice of cork and to refer to these microscopic units as “cells” [1]. Likewise, Antonie van Leeuwenhoek (1632–1723) initially described blood cells, skeletal muscle fibres, epithelial cells, teeth and circulatory system structures, using his handcrafted microscopes [1]. Thus, in vitro cultivation of animal tissues became possible in 1882, when Sydney Ringer developed the first balanced salt solution, the Ringer’s solution, whose composition closely resembled that of bodily fluids, and successfully kept frog hearts beating after dissection and removal from the body [2]. In 1885, the availability of saline solutions allowed the zoologist Wilhelm Roux to keep chicken embryonic cells alive for a few days, reporting the first example of in vitro cell culture [1].

**Figure 1:**
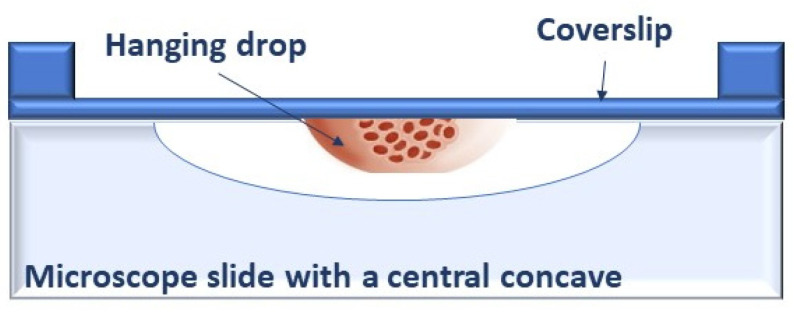
Schematic diagram of the hanging drop technique proposed for the first time by Harrison in 1907 [3]. In this method, a droplet of medium containing suspended cells (shown as dark red spots) is placed on a coverslip that is inverted to allow the falling of the drop in the concave well of the microscope slide. Cells aggregate due to gravitational forces and finally form spheroids.

Pioneering techniques of tissue culture were further proposed in 1907, when Ross Granville Harrison successfully monitored the outgrowth of nerve fibres from small pieces of frog embryonic tissue, which were maintained outside the body in the presence of lymph fluid freshly drawn from the lymph sacs of an adult frog [3]. Harrison placed the frog tissue on a coverslip in a solution of lymph and then inverted the material on a glass slide with a depression in it. As a result, the explanted tissue was maintained in a hanging drop (Figure 1) [3]. Nowadays, analogous protocols are still used to establish scaffold-free 3D cultures via the hanging drop technique [4]. In the same years, Alexis Carrel and Montrose Thomas Burrows greatly improved cell culture technologies by effectively cultivating chicken embryonic cells, and later mammalian cells as well [5,6].

However, somatic cells derived from animals typically died after a definite number of divisions, imposing fresh cell preparation for each experiment. A turning point for cell culture advancement was 1943, when Wilton Robinson Earle generated the first continuous cell line, the “L” cell line, from subcutaneous mouse tissue [7]. A few years later, in 1951, George Otto Gey established the first human immortal tumoral cell line, the HeLa cells, derived from cancerous tissue of the cervix of Henrietta Lacks, a 30-year-old mother of five, who died of cervical cancer on 4 October 1951 [8]. More than 80,000 scientific studies using HeLa cells have been published since the 1950s, and these cells have been used to study every possible aspect of cellular physiology as well as the basic machinery common to all cells, allowing many ground-breaking advances in science and medicine [9].

The broad use of HeLa cells in many labs around the world and their ability to indefinitely grow and overwhelm other cell lines have led to more than 300 different HeLa progeny strains and to a widespread cross-contamination of other cell lines [9,10]. It has been estimated that about 20% of cell lines are falsely identified (mainly due to intraspecies contamination), highlighting the need for proper authentication of the true origin of cell lines, as already suggested by the pioneering work of Walter Nelson-Rees in the 1970s and early 1980s [11]. Different strains cultured in different labs show different genomic and transcriptomic landscapes, mainly due to genotypic drift and accumulation of genetic aberrations due to the extensive chromosome instability characterizing cancer cell lines [10,12,13]. Thus, cancer cells are neither clonal nor genetically stable, and the same experiment performed on cells cultured in different labs could lead to distinct conclusions and irreproducible results. This variability is especially dangerous when preclinical studies on drug sensitivity are performed.

Advances of single-cell technologies have recently allowed the profiling of human tumours, showing that most of them are composed of genetically and phenotypically heterogeneous cell populations [14]. This internal tumour heterogeneity, consisting of a large number of mutations and chromosomal changes, but also dependent on epigenetic regulation, is regulated in vivo by a complex network of still unknown mechanisms, including tumour evolutionary trajectories, microenvironmental factors and the pre-existence of genetically distinct resistant cells [14]. At variance, heterogeneity in in vitro cell cultures mostly derives from the accumulation of stochastic mutations and chromosomal changes [12,13,15] that do not necessarily overlap with in vivo events. Thus, immortalized cancer cells cannot help researchers to address the cellular diversity arising in cancer tissues during the progression of the disease. In addition, when cultured in vitro, cancer cells miss interactions with the extracellular matrix (ECM) and neighbouring cells. Hence, their microenvironment is far away from the in vivo peritumoral niche, where different types of cells interact with and receive signals from other adjacent cells. Finally, the absence of interactions with other types of bordering cells and ECM components may support, in cells cultured in planar 2D monolayers, the loss of the differentiated phenotype commonly shown in vivo by those cells.

The non-physiological morphology and interactions that characterize cells in 2D cultures impact a variety of cellular processes, including proliferation, differentiation, and cell death. When tumour cells grown in 2D or 3D cultures have been compared, evident differences appeared not only in their morphological aspect but also in crucial biological properties, such as gene/protein expression, growth rate and invasive behaviour [16,17,18,19,20]. Notably, important discrepancies have been found between 2D- and 3D-cultured cells in their sensitivity to either targeted or classical chemotherapy drugs [21,22]. As an example, 3D cultures of breast-cancer-derived cell lines were more resistant to both HER-targeted (neratinib) and conventional chemotherapy (docetaxel) drugs when compared to 2D cultures [16]. More recently, a different drug sensitivity between colorectal cancer cells grown in 2D or 3D systems was observed for the pyrimidine analog 5-fluorouracil and the EGFR-blocker erlotinib [22]. Again, head and neck squamous cell carcinoma (HNSCC)-derived cells grown in 3D showed decreased sensitivity to either the alkylating agent cisplatin or the EGFR-targeted drug cetuximab [20]. The different pharmacological responses observed in 2D and 3D systems could be responsible at least in part for the high failure rate associated with drug discovery, where most preclinical cell-based screenings are carried out on 2D-cultured cells. It has been estimated that about 90% of successful cancer treatments tested preclinically fail in the early phases of clinical trials, and less than 5% of oncology drugs are effective in clinical trials [23].

In the next sections, we will summarize whether and how some of these limitations can be overwhelmed by the application of 3D cancer cell cultures. We will especially discuss the biological significance of (*a*) cell–ECM interactions and (*b*) cell–cell contacts in 3D systems that better mimic the complex microenvironment of tumours, thus providing more representative and therapeutically relevant models. Special emphasis will be placed on 3D co-culture models in which two or more different populations of cells, i.e., cancer, endothelial and/or other tumour-associated cells, are grown with some degree of contact to further increase the predictive value of 3D models.

## 2. Back to the Past: Restarting from 3D Spheroids

Scientists cultured cells in 2D monolayers for nearly a century before turning back to 3D cultures, as originally proposed by Harrison at the beginning of the 20th century [3]. The first observation of the ability of dissociated cells to create aggregates, i.e., 3D structures, dates back to 1957 [24]. The term “spheroids” was later coined by Robert Sutherland in the early 1970s to describe 3D structures formed by Chinese hamster lung cells when grown in suspension in spinner flasks [25]. Since then, spheroids have been generated from many primary cell types and cell lines in a multitude of approaches—hanging drop technique, low cell attachment plates, micropatterned surfaces, rotating bioreactors—that can be basically categorized into scaffold-free or scaffold/matrix-based [26]. However, it is important to emphasize that the term spheroid should be exclusively used for compact and stable cell aggregates with a spherical shape that can be manipulated or relocated without disintegration. Loose packages of cells that aggregate but do not form tight structures must not be misleadingly defined as spheroids. In addition, tumour cells that cannot form spheroids in vitro have been described, such as the breast cancer cell line SK-BR-3 and cell lines growing in suspension [27]. The ability of cells to aggregate into spheroids depends on the formation of homotypic adherent junctions that is mediated by cellular adhesion molecules such as E-cadherin in epithelial cells and tumours. A different expression of E-cadherin has been associated with altered spheroid formation in head and neck carcinoma cell lines, with cells expressing low levels of the protein unable to form tight spheroids [28]. In addition, the expression of other cell adhesion molecules may affect spheroid formation in colon cancer cells [29]. Notably, intracellular molecules involved in the stabilization of adherent junctions, such as α-catenin linking cadherins to cytoskeleton, are also required for spheroid formation. Indeed, cells lacking α-catenin are unable to tightly associate, despite cadherin expression [30].

**Figure 2:**
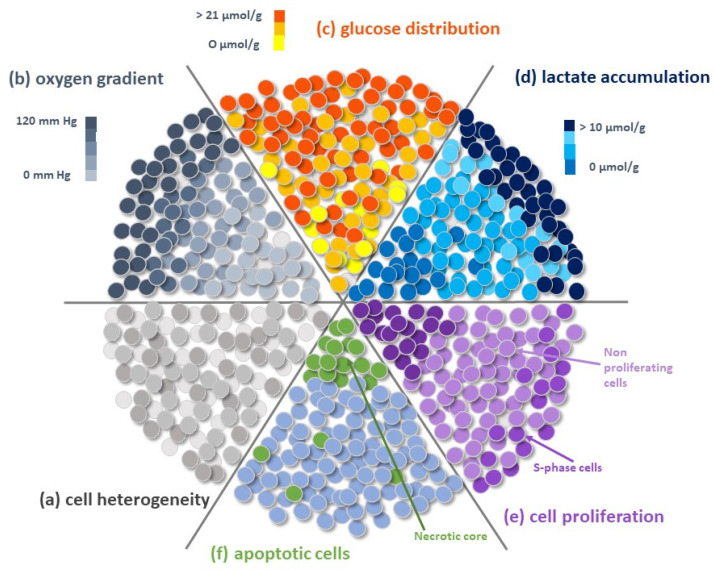
Schematic representation of a spheroid. (**a**) Spheroid heterogeneity, due to the presence of cells with different gene expression profiles and phenotypes, is represented by different grey spots. (**b**,**c**) The spheroid is composed of functionally differentiated layers resulting from the impaired distribution of oxygen ((**b**), dark grey scale) and nutrients, mainly glucose ((**c**), orange-yellow scale) that typically decrease from the periphery to the core of the spheroid. Consequently, the spheroid shows a well-defined spatial architecture characterized by the different distribution of (**d**) metabolites, i.e., lactate (blue-azure scale); (**e**) proliferating cells (violet scale) with the more dividing cells (S-phase cells) in the external layers and quiescent cells at the centre of the spheroid; (**f**) apoptotic cells (azure-green scale) that accumulate in the spheroid hypoxic core.

**Figure 3:**
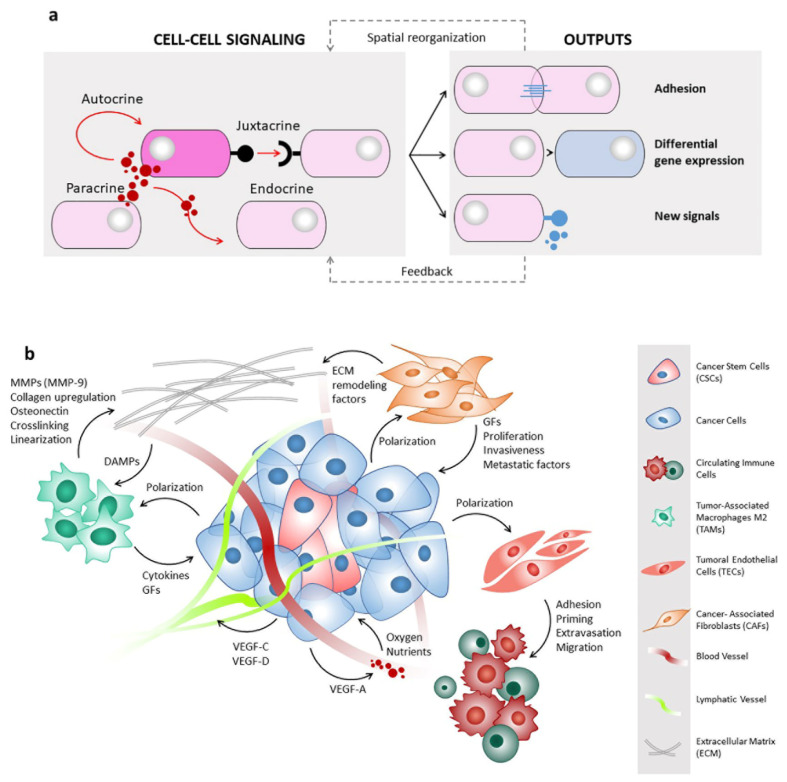
Types of cell–cell interactions and communications in tumour microenvironment. (**a**) Cells communicate through various types of signalling that allow chemicals to travel to target sites in order to elicit a biological response. In autocrine signalling, cells produce ligands that induce a cellular response through cognate receptors for those molecules expressed in the same cell. Paracrine signalling depends on the local diffusion of secreted molecules from one cell to other neighbouring cells. In contrast, cell–cell contacts are required for iuxtacrine signalling, where molecules directly pass between cells via gap junctions or other structures without secretion into the extracellular space. In endocrine signalling, molecules are secreted and travel long distances through extracellular fluids such as the blood plasma to reach target cells and tissues; typical mediators of this communication are hormones. The action of signalling molecules on target cells triggers new downstream signalling, modifies gene expression, and influences cell–cell adhesive properties and spatial organization. In response to these changes, feedback mechanisms can be activated to maintain cell/tissue homeostasis. (**b**) Multiple cell types are present in the tumour microenvironment, and all of them contribute to the maintenance of the complex and dynamic network of signalling molecules characterizing the peritumoral niche. Tumours and cancer stem cells (CSCs) produce angiogenic and lymphangiogenic factors (VEGF-A and VEGF-C/D, respectively), and in turn, blood vessels deliver nutrients and oxygen. In addition, tumours and CSCs secrete molecules able to induce the acquirement of a pro-tumoral phenotype, i.e., able to polarize macrophages to M2 subtype, fibroblasts to CAFs, and ECs to TECs. All these cell types drive cancer cell proliferation, invasion, and metastasis through the production of growth factors, cytokines, and ECM remodelling enzymes.

The spherical geometry of spheroids is characterized by an external proliferating region and an internal quiescent zone surrounding a necrotic core (Figure 2). This organization depends on the gradient of nutrients and oxygen diffusion and simulates the cellular heterogeneity typical of solid tumours. This “metabolic zonation” also mirrors different metabolic activities among tumour cells placed in different layers [31]. Thus, the unique cyto-architecture of spheroids mimics in vivo cell morphology, proliferation, oxygenation, nutrient uptake, waste excretion and drug uptake and allows the maintenance of cell–ECM interactions and signalling, thereby regulating molecular functions and cellular phenotypes [32,33,34,35]. The presence of ECM is crucial for controlling key parameters of spheroids, such as pH, oxygen and nutrient concentration gradient, cell morphology and size [36,37]. In addition, spheroids can be single-cell spheroids, including a single type of cells, i.e., cancer cells, but can also be multicellular spheroids [38], in which two or more different populations of cells are combined in the attempt to reconstitute the tumour micro-environment (TME) surrounding tumours in vivo, which includes stromal cells (fibroblasts, endothelial cells, immune cells, mesenchymal stem cells), other non-malignant host-tissue cells, and blood and lymphatic vessels, all embedded in the ECM (Figure 3).

## 3. Cell–ECM Interactions in 3D Spheroids

The ECM is a dynamic matrix—mainly composed by collagens, proteoglycans, and glycoproteins—that provides not only physical support for the cellular constituents but also initiates biochemical and biomechanical signals involved in tissue morphogenesis, differentiation, and homeostasis. The active role of ECM in cellular physiology was first proposed by Mina Bissell, who showed the impact of ECM on gene expression [39]. Molecules of the ECM can bind and sequester soluble growth factors and other ECM-associated proteins to protect growth factors from degradation or to develop concentration gradients, thus controlling cell growth and directing cell migration within the tissue. In addition, ECM components can interact with cell surface receptors, such as integrins and receptor tyrosine kinases. The crosstalk between integrins and growth factor receptors directly regulates downstream cell signalling and growth factor–induced biological processes, mainly proliferation and invasion [40].

All the ECM components are post-translationally modified by an array of cell-secreted remodelling enzymes and undergo continuous adapting processes as a result of the interplay among deposition, modification, degradation and physical organization. Collectively, ECM remodelling affects the 3D spatial topology of the matrix surrounding cells and finally their biochemical and biophysical properties. The process of ECM remodelling is tightly regulated during development, as well as in tissue homoeostasis during wound repair. At variance, a dysregulated remodelling is associated with pathologic conditions, such as inflammatory diseases, tissue fibrosis and cancer [41]. The establishment of a cancer-supporting matrix that actively contributes to the pathogenesis of tumours depends on multiple mechanisms and players. Tumour cells themselves can express altered levels of both ECM components and/or modifying enzymes. The major producers of ECM in cancer tissues are, however, fibroblasts, especially the so-called cancer-associated fibroblasts (CAFs), that derive from the local differentiation of stromal cells via tumour-derived activation factors [42].

### 3.1. Matrix Stiffness

The deposition of major ECM components, such as fibrillar collagen, favours the cross-link of collagen fibres via the activity of ECM-modifying enzymes, thus increasing matrix stiffness. Matrix stiffness strongly influences the phenotype and epigenetic landscape of tumour cells by altering physical forces and mechano-signalling [43]. In general, an increase in matrix stiffness promotes interactions between ECM macromolecules and tumour cell surface receptors, triggering integrin-mediated signalling and cell proliferation [40,44]. In patients, increased tumour/stroma stiffness is a marker of disease progression and poor prognosis [45], as confirmed in various solid tumours such as breast, pancreatic and colon cancers [46,47,48]. Notably, an increase in the density of breast tissue is linked to an enhanced risk of breast carcinoma, thus supporting the benefit of the mammographic screening for the prevention of breast tumours [46]. In addition, the dysregulated clustering between integrin and growth factor receptors induced by stiffness may result in drug resistance, as shown for the HER2 inhibitor trastuzumab in HER2-positive breast cancer cells [49].

More complex relationships exist between matrix stiffness and 3D migration, wherein cells can move using either proteolytic (mesenchymal) or nonproteolytic (amoeboid) strategies [50,51]. In proteolytic migration, cells move throughout cavities generated by the enzymatic breaking down of ECM macromolecules due to the activities of cell-secreted or cell-membrane-linked proteases. Otherwise, certain cell types, such as inflammatory or tumour cells, can utilize amoeboid migration that allow them to overcome the biophysical matrix resistance by forcing through the ECM, or deforming it, independently of proteolysis. Thus, 3D cell migration is the net result of proteolysis-dependent and -independent mechanisms. Overall, cells encapsulated within relatively stiff gels require proteolytic activity to migrate, whereas a slight decrease in matrix cross-linking density allows protease-independent cell migration [52,53]. The matrix elasticity of the peritumoral microenvironment is therefore a critical modulator of cancer invasion and metastasis. Remarkably, it has been proposed that the invasive behaviour of tumour cells increases when matrix stiffness is closer to that of the organs where in vivo metastasis is more frequently observed [54]. The in vitro design of 3D matrix composition and stiffness is therefore crucial to faithfully reproduce in vivo environments.

Remarkably, appropriate matrix conditions can allow invasion of cancer cells but not of normal cells. In this way, the matrix can selectively capture tumour cells, taking advantage of their tendency to undergo rapid and non-proteolytic ameboid migration during the early stage of invasion. In fact, selective gelatinous micro-fibrous gel matrices with specific elasticity have been established to permit invasion by malignant cancer cells, and in some cases, cytotoxic drugs have been loaded into the matrix to exert an anticancer effect only on the invading tumour cells [55,56]. These results suggest that 3D models may represent a valuable tool for the design of innovative drug carriers based on the modulation of matrix stiffness to selectively capture tumour cells.

In addition to stimulating survival pathways and influence invasiveness, an increased matrix stiffness can also foster resistance to chemotherapy and targeted drugs [57]. The ECM collagenous scaffolds can absorb anticancer drugs, thus affecting their delivery, and the deposition of ECM components can increase the density of spheroids and create a physical barrier that reduces drug penetration [58].

### 3.2. Matrix Remodelling

Tumour and stromal cells commonly express increased levels of ECM-degrading proteases, such as metalloproteinases (MMPs), disintegrin and metalloproteinases (ADAMs), disintegrin and metalloproteinases with thrombospondin motifs (ADAMTS), and proteases that specifically cleave at serine, cysteine, and threonine residues. Conflicting results on their pro- or anti-tumorigenic properties have been reported [41], suggesting a context-dependent effect of ECM-degrading enzymes. As an example, overexpression and high serum levels of MMP-8 have been correlated with decreased survival in patients with solid cancers such as ovarian cancer, hepatocellular carcinoma and colon carcinoma [59]. In contrast, the absence of MMP-8 increased susceptibility to chemically induced skin tumours in mice, and bone marrow transplants of MMP-8-expressing neutrophils restored tumour protection [60]. However, the establishment of a cancer-supporting matrix requires the proteolytic degradation of normal ECM components and their replacement with tumour-derived molecules favouring cancer progression [41]. The degradation of ECM is also required to sustain proteolytic cell motility (see above). In addition, ECM components bind and sequester soluble growth factors, thus maintaining them in an inactive form. Fibronectin, for example, binds the fibroblast growth factor-2 (FGF-2) and the vascular endothelial growth factor-A (VEGF-A) [61]. The enhanced protease activity of matrix-associated enzymes causes growth factor release, making them able to bind and activate their receptors in surrounding tumour/stromal cells to induce growth and/or invasion [41]. Crucially, the paracrine action of FGF/VEGF on the peritumoral endothelial cells (ECs) promotes neo-angiogenesis [62].

The cleavage of ECM components further produces bioactive fragments, termed matrikines for their structural similarity to chemokines or cytokines. These fragments can be endowed with pro- or anti-tumorigenic properties and are important regulators of the angiogenic switch [62]. For example, matrikines derived from the degradation of elastin via elastases and MMPs commonly promote tumour progression, whereas some collagen-derived fragments, such as endostatin, inhibit angiogenesis [63,64].

### 3.3. Matrix and Immune Cells

The ECM remodelling activities induced by tumour and stromal cells promote the establishment of an inflammatory TME. ECM-degrading proteases can release ECM components, known as danger-associated molecular patterns (DAMPs), that directly act as inflammatory stimuli by inducing immune responses through the interactions with pattern recognition receptors (PRRs) expressed by immune cells [65]. Matrikines may also function as DAMPs in tumours.

In addition, bone-marrow-derived cells, such as tumour-associated macrophages (TAMs) and tumour-associated neutrophils (TANs), are an important source for ECM remodelling proteases in the primary tumour environment [66,67]. Neutrophils are recruited to the tumour site by hypoxia and constitute the main source of angiogenesis-inducing MMP-9. TAMs foster a pro-tumorigenic environment, especially when polarised to an M2-like phenotype, by inducing proteolytic clearance and degradation of interstitial collagen. TAMs may contribute to ECM deposition by upregulating collagens’ synthesis and inducing crosslinking and linearization of collagen fibres that favour tumour invasiveness [68]. Macrophages also deposit osteonectin, a glycoprotein that promotes stromal invasion in a mouse model of breast cancer [69].

In conclusion, the ECM is a major component of the TME, and coordinates cellular processes, including proliferation and invasion, that are highly dysregulated in cancer. Collectively, the net effect on tumour growth, invasiveness and vessel formation depends on the composition/stiffness of the ECM matrix, the context-dependent activity of ECM-degrading enzymes, and the receptors expressed in tumour and stromal cells. In addition, a detailed knowledge of the levels of expression of genes encoding ECM components and ECM-associated proteins may propose these molecules as novel biomarkers and therapeutic targets. All these factors are likely tissue-/tumour- and patient-specific, but they are also expected to be highly dynamic during cancer progression, thus offering a constant evolving picture of the peritumoral ECM during the onset and development of the disease. In fact, computational and proteomic technologies have allowed the characterization of the ECM of various tumours and microenvironmental niches [70]. These approaches have permitted the description of specific protein signatures, termed “matrisome”, able to distinguish tumours from normal tissues, tumours of different stages, primary from secondary tumours and tumours from other diseased states such as fibrosis.

## 4. Cell–Cell Communications

Cell–cell interactions regulate development, tissue homeostasis and single-cell functions [71]. Tumours and their surrounding microenvironments are complex communities where different subsets of cells interact and generate physical and biochemical signals that are sent between and/or within cells to induce intracellular effects (Figure 3). Studying cell–cell communications within these communities may reveal how cells communicate in these ecosystems and support the development of effective prognostic markers and/or innovative therapeutic targets.

### 4.1. Cancer-Associated Fibroblasts (CAFs)

CAFs are major components of tumoral stroma, wherein they actively communicate with cancer cells to sustain their proliferation, invasiveness, and metastatic properties [42]. CAFs also have a central role in the remodelling of ECM and in the control of volume, composition, and stiffness of the tumour stroma. Therefore, studies on cell–cell interactions focused on the crosstalk between tumour and stromal cells are needed.

Outstandingly, CAFs do not communicate with other cell types in the TME merely via paracrine signalling but also by direct physical contact. For example, the interaction between squamous cell carcinoma (SCC) cells and CAFs was required during SCC cell migration in a 3D invasion model [72]. In this culture system, SCC cells were grown on the top of a matrix mainly composed of fibrillar collagen I. The presence of CAFs into the matrix caused SCC cell lines to invade. A multi-photon confocal time-lapse imaging of the 3D cultures demonstrated that SCC cells invaded in close proximity to fibroblasts, appearing to move “along” them. Very interestingly, CAFs were the leading cells in each migratory pathway, where they created tracks for the invasion of SCC cells, which were unable to migrate independently [72]. Again, A549 lung cancer cells migrated through a synthetic vessel seeded with fibroblasts in a microfluidic model [73]. The requirement of direct cell–cell contacts was confirmed by the observation that conditioned media from tumour-derived CAFs did not enhance the invasion of colon cancer cells, but the physical presence of CAFs in mixed 3D spheroid was required to induce cell invasion [74].

The role of CAFs in promoting cancer progression has been studied in different types of cancers, including prostate cancer. When androgen-dependent prostate cancer cells have been co-cultured with CAFs in mixed 3D spheroids, their sensitivity to anti-androgen drugs was reduced [75]. Similarly, the response of 3D-cultured tumours to radiotherapy [76] and the resistance of spheroids from colorectal carcinoma cell lines to clinically relevant drug combinations [22] were both modified by the addition of CAFs, supporting the theory that the presence of CAFs promotes drug resistance.

Cell-type-specific differentially regulated genes, indicative of activated stroma, have been detected in breast and non-small-cell lung cancer [77,78]. More recently, by comparing transcriptome of CAFs isolated from human ovary cancers, two distinct signatures have been identified, suggestive of CAF heterogeneity in the TME. In addition, different signatures of CAF are differently associated with patients’ survival rates, implicating that either tumour cell or CAF subtype signatures may have prognostic and predictive values in ovarian cancer [79]. Interestingly, a multicellular computational systems biology platform, termed Cell–Cell Communication Explorer (CCCExplorer), has been experimentally validated to identify paracrine/autocrine tumour–stroma interactions and to uncover novel crosstalk targets and drug candidates [78].

Together, these studies suggest the complexity and heterogeneity of CAF–cancer cell interactions. The requirements for paracrine signalling and/or direct contact may vary for different functions in different tumours or even cell types. The chance of studying the CAF–tumour cell interactions in multicellular 3D systems, mirroring the complexity of the TME, may help to enhance knowledge on cancer biology and to set up improved models for pre-clinical in vitro studies on drug discovery.

### 4.2. Immune Cells

Infiltration by immune cells is characteristic of most forms of malignancy, and macrophages are the most abundant immune/inflammatory cells present in the peritumoral niche. Tumour-associated macrophages (TAMs) derive from circulating monocytes recruited by tumour-generated signals and can be phenotypically polarized by the microenvironment to activate M1- or M2-specific programs [80]. This functional plasticity results in the acquisition of either anti- or pro-tumoral properties. In general, M1 TAMs show anti-inflammatory properties in the early stages of cancer, but they polarize towards the M2 phenotype as the tumour develops [80]. M2 TAMs secrete cytokines and growth factors, thus favouring inflammation [80]. In addition, M2 TAMs directly promote invasion and ECM remodelling by the expression and activation of MMPs, particularly MMP-9, which releases matrix-bound pro-angiogenic cytokines, such as VEGF, and growth factors that promote cancer cell proliferation [80]. The pro-tumoral activity of M2 TAMs makes them a suitable target for anti-tumor therapies [81,82]. TAMs are also drivers of an immunosuppressive TME through the secretion of anti-inflammatory cytokines, which inhibit the survival of cytotoxic T lymphocyte and recruit immunosuppressive regulatory T cells [80]. However, most of our present knowledge is based on rodent models, and species-specific differences in TAM recruitment and activation mechanisms have been reported [81]. Thus, better models are needed to increase our insight into the role of TAMs in the TME.

Macrophages can be included in multicellular tumour spheroids by directly forming heterogeneous spheroids from a mixed macrophage/cancer cell suspension. However, interestingly, macrophages have been reported to spontaneously infiltrate tumour spheroids, thus offering a more physiologically relevant method of macrophage inclusion. Interestingly, macrophages displayed tumour-promoting properties only when incorporated with cancer cells in mixed spheroids. Compared with models in which macrophages were embedded in the surrounding matrix, mixed spheroids from breast cancer cells showed an increased expression of M2-associated cytokines, a faster oxygen consumption and resistance to the cytotoxic drug paclitaxel [83]. In addition, when infiltrated by macrophages, breast cancer cells switched from an MMP-dependent mesenchymal migration to an amoeboid mode resistant to protease inhibitors [84]. Again, a direct contact between tumour cells and macrophages is required, suggesting that, in addition to paracrine mechanisms, direct cell–cell interactions are crucial for modulating the behaviour of both tumour and macrophages in the TME.

In general, clinical studies have shown a positive correlation between TAM accumulation and poor patient prognosis for multiple cancer types, including brain tumours, especially glioblastoma (GBM), where TAMs are the most abundant non-neoplastic cell type, including up to 40% of the total cells [85]. Knowledge about TAM phenotypes and functions in GBM is, however, still scarce, and the setup of heterogenous models should be very useful to deeply investigate TAM/GBM cell dynamics [85,86]. Mechanics and compositions of brain tissues are, however, quite different from other tissues, e.g., breast tissues, that display a higher abundance of fibrillar collagens. Accumulating evidence suggests that matrix composition, stiffness and cross-linking density, as well as matrix-mediated signals, all influence macrophage polarization and phenotype [87]. Gene expression profiles resembling immunosuppressive TAMs have recently been shown to be induced by high-density collagen in macrophages [88]. This mechanism could decrease the efficacy of immunotherapy and elucidate why high collagen density is frequently linked to poor tumour prognosis.

The study of the interactions between cancer and immune cells in the peritumoral niche has elucidated the ability of tumours to modify local immune cell functions to their advantage. The TME is indeed able to prevent the expansion of tumour antigen-specific helper and cytotoxic T cells and to promote the production of proinflammatory cytokines, leading to the accumulation of suppressive cell populations that inhibit instead of promote immunity. These observations have opened the way to immune oncology, a ground-breaking pharmacological approach for the cure of tumours, based on the enhancement of tumour immunity through the block of inhibitory pathways and inhibitory cells in the TME via immune checkpoint inhibitors. Due to the increasing clinical use and resistance to immunotherapies [89], it is crucial that relevant components of the immune system start being incorporated into 3D co-culture models to better predict clinical outcomes.

### 4.3. Endothelial Cells

The development of heterogeneous, chaotic, distorted and leaky blood vessels accompanies and supports the development of solid tumours [90]. The new vessels supply the growing tumour with oxygen and nutrients and facilitate cancer cell intravasation. Neo-angiogenesis can be induced via angiogenic factors, such as VEGF-A, commonly secreted by tumour cells [91]. Similarly, the formation of lymphatic vessels can be induced by tumour-derived VEGF-C and VEGF-D [92].

A growing body of evidence suggests that the ECs lining peritumoral vessels, termed Tumour Endothelial Cells (TECs), exhibit unique phenotypic and functional properties when compared to normal endothelial cells. They are highly proliferative and exhibit genetic instability and a stemness gene signature [93]. Furthermore, TECs not only promote tumour angiogenesis but also impact the immunogenic properties of the peritumoral niche. TECs can actively guide adhesion, rolling and extravasation of circulating immune cells into the tumour stroma, and impact T lymphocyte priming and migration [94]. In addition, TECs directly affect cancer progression and the formation of distant metastasis through angiocrine and paracrine signalling [93]. The leaky architecture of tumour vessels facilitates the hematogenous metastatic spread of cancer cells [95], and the adhesion molecules expressed by TECs act as scaffold directing malignant cells to intravasation [96]. Moreover, TECs use MMPs to break down the vessel basement membrane, thus directly impacting metastasis [97]. In addition, ECs of cancer-associated lymphatic vessels and their VEGF-C-mediated signalling are critical for lymphatic metastasis [98].

In the last few years, various models to study the reciprocal crosstalk between cancer cells and vascular ECs have been proposed. In these systems, common readouts are cancer cell migration and EC sprouting as indexes of early stages of metastasis and tumour angiogenesis. In general, when tumour cells were co-cultured with ECs, cancer cell migration toward ECs was enhanced when cells were maintained in close proximity [99,100,101]. On the other hand, ECs underwent sprouting and formed blood-vessel-like structures able to surround tumour spheroids. Furthermore, when embedded in mixed spheroids, ECs may undergo the endothelial-to-mesenchymal transition (EndMT) process, in which ECs lack endothelial markers and properties to acquire a mesenchymal/fibroblastic phenotype with higher invasive and migratory potential. The increase in the fibroblastic component significantly increased the density of spheroids that may develop resistance against targeted and cytotoxic therapies [102].

In most 3D heterogeneous models, human umbilical vein ECs (HUVECs) were used as EC paradigm. Actually, in recent years, organ specificity and function of the endothelium and ECs have been clearly demonstrated [103]. Similarly, the regulation of vascular niches is likely to be organ-specific. From this perspective, HUVECs derive from a rather unique tissue with distinctive properties and are quite different from TECs lining the TME microvasculature [93,104]. Therefore, 3D models including HUVECs do not strictly reflect organotypic tumour-vasculature crosstalk. These aspects are especially crucial when brain tumours, such as GBM, were studied. Brain ECs are indeed of microvascular origin and are strictly interconnected with GBM cells [105]. The highly invasive nature of GBM is mediated via the spread of cancer cells along the vasculature, and an increased blood vessel density is a hallmark of GBM. Moreover, the self-renewal and maintenance of the subpopulation of cancer stem cells (CSCs) present in GBM are critically related to the physical association of CSCs with blood vessels in perivascular niches [106]. In turn, CSCs can stimulate the formation of new blood vessels due to their proangiogenic capabilities [107]. The setup of 3D systems including CSCs/GBM cells and brain ECs would be beneficial to address the molecular and cellular mechanisms that regulate the impact of brain ECs on GBM stemness, invasiveness and relapse and conversely the mechanisms of neovascularization utilized by GBM cells [108].

Finally, it should be considered that cellular sex has rarely been studied as a biological variable in preclinical research, even when the pathogenesis of diseases with predictable sex differences is studied. Solid epidemiological data indicate that sex differences exist in the incidence of cardiovascular disease, disorders of the immune system, depression, addiction, asthma and cancers, including GBM [109]. Male and female GBMs are biologically distinct, and improving outcomes may require sex-specific approaches to treatment. Similarly, sex-dependent differences have been described in ECs from different vascular beds [110,111,112,113,114,115]. Thus, sex as a biological variable should be taken into account to reconstitute a more physiologically relevant in vitro TME.

## 5. Beyond Multicellular Spheroids

It is still evident the constant need of developing new in vitro experimental models able to engage the whole TME components and interactions (e.g., matrix compositions and stiffness, types of cells with direct or paracrine contacts) that finally regulate the capability of tumours to growth, metastasize, skip immune regulation, and acquire drug resistance. In the following paragraphs, we will briefly introduce some of the most updated approaches designed to overcome the current limitations in the study of the crosstalk between tumour cells and other components in the TME.

### 5.1. Engineered Metastatic Niches

Synthetic metastatic niches represent an emerging technology for the analysis of the metastatic microenvironment, providing a valid approach to study extravasation and colonization of metastatic sites and the molecular mechanisms of metastatic cancers [116]. Since the engineered niche largely phenocopies a natural metastatic niche, this platform may also be useful to identify novel tumoral markers and pharmacological targets [117]. Engineered metastatic niches include infiltrating cells that contribute to the formation of connective tissue, the vascular network and immune cells and could be entirely synthetic implants or cell-laden sites that require ex vivo culture prior to implantation [116]. Thus, synthetic niches are able to mimic many aspects of native metastatic niches, with the advantage of being able to tune the metastatic microenvironment [116,117,118]. Because of its plasticity, this method provides a useful platform to study the interactions between disseminated tumour cells, stroma and immune system cells. Indeed, bioengineered niche composition can be recreated in vitro to reproduce in vivo sites where tumour cells preferentially metastasize [117,118]. Therefore, they can be built to enrich or deplete specific factors, to study tumour cell recruitment and phenotypes of invading cells and to examine the role of specific cell types in the niche.

### 5.2. Cancer-on-Chips

**Figure 4:**
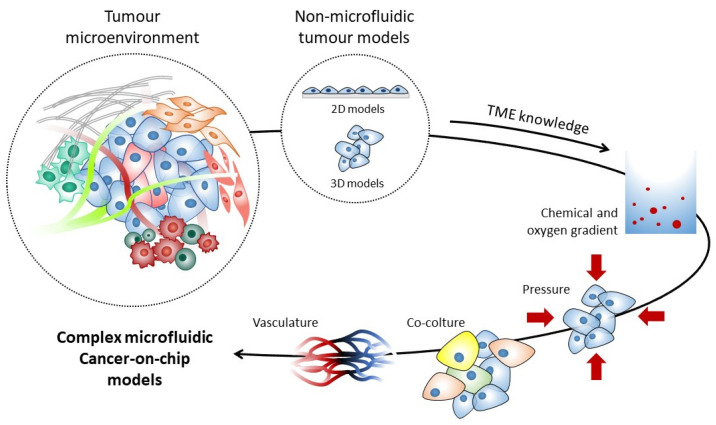
The microfluidic cancer-on-chip model. The setup of models suitable for the study of the complex interactions among different cell types and signalling molecules within the tumour microenvironment is still a major challenge. Commonly used 2D in vitro models poorly represent in vivo tumoral niches, and thus many 3D models have been developed. Among these models, the microfluidic cancer-on-chip model has the unique advantage of recapitulating tumoral microenvironment in a bioengineered microdevice able to summarize crucial functional aspects of the peritumoral niche, e.g., gas exchange, to maintain chemical and oxygen gradients, the preservation of biomechanical forces and pressure, the inclusion of multiple cell types and finally the presence of endothelial cells organized in an aligned structure resembling blood vessels.

Organs on chips (OoC) are bioengineered microdevices that recapitulate key functional aspects of organs and tissues [119]. All OoC platforms have three main characteristics: (a) the 3D nature and arrangements of tissues on the platforms; (b) the presence and integration of multiple cell types (such as parenchymal, stromal, vascular and immune cells); (c) the presence of biomechanical forces relevant to the tissue (for example, hemodynamic shear stress for vascular tissues). Specifically, cancer-on-chip (CoC) models exploit microfluidic systems to build platforms able to mimic cancer and stromal cell interactions in the TME [120,121] (Figure 4). CoC models are typically a few centimetres size and include some basic components, namely a microfluidic chip equipped to control fluid flow, matrix materials, cancer cells and other optional cell types. Each type of chip has different controlled parameters and read-outs, commonly based on the tracking of cell and invasive lesion, gradient sensing, staining and gene expression quantification. Furthermore, CoCs are designed for different cell culture options: (a) 2D chip, in which cancer cells are exposed to a gradient of a solute to study cancer cell viability or migration [122,123]; (b) lumen chip, where a 3D scaffold is used to form lumens or tumour compartments, typically used to model blood tumour vessels [124,125]; (c) compartmentalized chip, the more versatile chip that permits both 2D and 3D cell culture and allows one to gather different types of matrix and cells in a controlled manner [126,127,128]; (d) Y chip, which resembles the compartmentalized chip, but is less versatile in its patterning possibilities [129]; (e) membrane chip, which is a multi-layered device useful for studying interactions between cancer cells and other cell types [130].

Typically, real-time imaging techniques in CoC models are used to analyse whether CAFs affect cancer cell migration. Thanks to these tracking techniques, it has been observed that cancer cell invasion is promoted when CAFs are co-cultured with tumour cells in a compartmentalized chip [100]. Using compartmentalized chips with cancer cells, macrophages and ECs, it has been shown that TAMs increase the ability of cancer cells to impair the endothelial barrier and intravasate [131]. Reciprocally, cancer cells can directly affect TAMs by increasing migration and polarization of resident macrophages in various CoC systems [132]. Several CoCs address the endothelial–cancer cell interactions, mainly using hydrogel matrices. These models are commonly characterized by a gel–fluid interface that is lined with ECs to mimic a vessel wall. Interestingly, these in vivo-like vessels have been designed to rely on EC self-assembly. These methods are very useful to observe dynamics of intravasation by testing the effects of different TEM compositions on EC resistance to cancer cell invasiveness [125,131].

Finding the most appropriate matrix, i.e., the choice of ECM components and stiffness, is a major challenge in CoC. The mechanical properties of ECM are indeed deeply involved in the regulation of cancer cell motility and invasion (see above). Injectable hydrogels, mainly collagen I and Matrigel, are often used as a 3D scaffold to support cell growth and migration in microfluidic devices, and stiffness and pore size of the matrix can be optimized by altering concentrations and ratios among its components [133]. Self-standing matrix layers, such as electrospun matrices, have been developed as an alternative support, offering more mechanical stability compared to hydrogels [134].

Interestingly, OoC and CoC have a great potential in precision medicine, allowing personalization of the platform with patient’s cells. Thus, OoC and CoC represent an innovative point of convergence between tissue engineering and precision medicine. Organoids are another type of multicellular 3D model reproducing some aspects of in vivo organ structure and function that are emerging as a promising platform for personalized medicine, especially for preclinical drug screening and to predict responsivity to selected therapies [135]. However, organoids are not classified as OoCs due to their production through stochastic self-organization (rather than specific cell seeding and growth protocols) and lack of cytoarchitectural structure (rather than scaffolding or specially shaped culture chambers).

## 6. Conclusions and Perspectives

The ability to correctly mimic the complexity of the TME is a main challenge for the development of physiologically relevant in vitro models for drug screening and fundamental cancer biology studies. Adequate pre-clinical models need to take into account either cell–ECM or cell–cell communication within and between the TME, thus requiring co-culture of multiple cell types in a defined 3D matrix. The availability of in vitro models that better phenocopied in vivo TME could thus offer the chance to identify chemical and physical microenvironmental factors involved in the early stages of tumorigenesis, and to discover still elusive molecular mechanisms that might be explored therapeutically. Finally, the design of customized synthetic niches and organ-on-chips from patient-derived tumour cells could allow the implementation of prevention, diagnosis and personalised therapies in the era of precision medicine.

## Data Availability

Not applicable.
